# Association of managerial position with cardiovascular risk factors: A fixed-effects analysis for Japanese employees

**DOI:** 10.5271/sjweh.3966

**Published:** 2021-08-31

**Authors:** Ryo Ikesu, Atsushi Miyawaki, Akiko Kishi Svensson, Thomas Svensson, Yasuki Kobayashi, Ung-il Chung

**Affiliations:** Precision Health, Department of Bioengineering, Graduate School of Engineering, The University of Tokyo, Tokyo, Japan; Department of Public Health, Graduate School of Medicine, The University of Tokyo, Tokyo, Japan; Department of Clinical Sciences, Lund University, Skåne University Hospital, Malmö, Sweden; Department of Diabetes and Metabolic Diseases, Graduate School of Medicine, The University of Tokyo, Tokyo, Japan; School of Health Innovation, Kanagawa University of Human Services, Kanagawa, Japan; Clinical Biotechnology, Center for Disease Biology & Integrative Medicine, Graduate School of Medicine, The University of Tokyo, Tokyo, Japan

**Keywords:** Key terms exercise habit, Japan, LDL, low-density lipoprotein cholesterol, longitudinal analysis, manager, self-reported sleep sufficiency

## Abstract

**Objectives:**

Although higher occupational classes have been reported to be associated with better health, researchers do not fully understand whether such associations derive from the position or individual characteristics of the person in that position. We examined the association between being a manager and cardiovascular disease (CVD) risk factors using unique panel data in Japan that annually observed employees’ occupational class and health conditions.

**Methods:**

We analyzed data for 45 888 observations from a Japanese company from 2013 through 2017. The association between being a manager and CVD risk factors (metabolic risks and health-related behaviors) were evaluated using simple pooled cross-sectional analyses with adjustment for age, sex, marital status, and overtime-working hours. We further incorporated employee-level fixed-effects into the models to examine whether the associations were subject to individual time-invariant factors.

**Results:**

The pooled cross-sectional analyses showed that, compared to non-managers, managers had 2.0 mg/dl lower low density lipoprotein cholesterol (LDL-C) level, 1.4 mmHg-lower systolic blood pressure, and 0.2 kg/m^2^ lower body mass index (BMI). After adjusting for employee-level fixed-effects, being a manager was associated with a significantly 2.2 mg/dl higher LDL-C level. However, the associations between an individual’s management status and blood pressure or BMI were not significant. Furthermore, managers were 5.5% less likely to exercise regularly and 6.1% less likely to report sufficient sleep in the fixed-effects models, although the pooled cross-sectional analyses did not demonstrate these significant associations.

**Conclusions:**

Our findings suggest the necessity of considering these unfavorable health risks associated with being promoted to a manager.

Health inequality among individuals who differ in socioeconomic status (SES) has been a critical concern for public health (1, 2). Previous studies have reported that higher SES (represented by higher household income, education level and occupational class) is correlated with better health status in terms of mortality and self-assessed health (3–6). Health disparity among occupational classes is drawing attention because of research examining stress in the workplace as a risk factor for cardiometabolic disease (7, 8). Evidence from European countries and the United States has consistently revealed that workers having a higher occupational status exhibit lower mortality and are in better health than those occupying a lower status (9–13).

When moving beyond correlation, however, it is unclear whether a higher occupational class has a positive net effect on an individual’s health. Some studies have argued for a cause-and-effect connection between higher occupational class and better health, possibly because of the potential for greater control and fewer psychosocial stressors for those in a higher occupational class. Nevertheless, previous cross-sectional or cohort studies have not accounted for the possibility that employees with some unobserved individual traits may have been more likely to be promoted (3–6, 9–13). For example, based on the famous Grossman model, those who have a lower time discount rate (ie, people who value the future) would spend more resources for their health and be more likely to invest in their skill development for promotion (14). Another study implied that the association between SES and health could be partly explained by individual traits such as tidiness (15). Dealing with these unobserved individual characteristics requires observing the same individuals as they experience changes in their occupational class over years.

Only a few studies have addressed this issue to date (16–19). Anderson & Marmot suggested the positive impacts of occupational promotion on heart disease (16). Other studies have shown that promotion to a managerial position is associated with poorer mental health, possibly due to job stress (17–19). Still, the association between occupational class and cardiovascular disease (CVD) risk factors has not been evaluated while adjusting for individual traits, even though the association between SES and CVD has been a major concern (9, 16, 20). A better understanding of this association should help policymakers and employers implement effective measures to protect employees from unfavorable health risks.

In this context, we aimed to examine the association between being a manager and CVD risk factors (metabolic risks and health-related behaviors) by using unique panel data that observed employees’ occupational class and health conditions annually in a large Japanese company. Judging from the possible association of promotion to a manager with job stress, we hypothesized that being a manager is associated with unfavorable CVD risk factors.

## Methods

### Study sample

We used unique panel data for a large Japanese company in the financial sector from 2013 through 2017. The panel data included basic registration data and data from an annual health checkup, which was required by the Japanese Government (21). The basic registration data comprised such variables as age, sex, marital status, occupational class, years of employment, and overtime-working hours. Medical claims data were also used for information on hospitalization. For our analyses, we included all employees observed in the longitudinal dataset during the period spanning fiscal 2013–2017. Of the 72 821 initial observations, we excluded (i) 4989 observations (6.9%) that were missing any variable in the registration data, or (ii) 22 375 observations (30.7%) for missing ≥1 of the following variables in the annual health checkup data: body mass index (BMI), abdominal circumference, systolic blood pressure, diastolic blood pressure, low-density lipoprotein cholesterol (LDL-C), high-density lipoprotein cholesterol (HDL-C), triglycerides (TG), fasting blood sugar, HbA1c, smoking status, exercise habits, and self-reported sufficiency of sleep. We excluded 26 933 observations [15 014 men and 11 919 women; mean age (SD) 34.0 (11.6) years at time of their exclusion]. Overall, we analyzed the data of 45 888 observations (63.0%) for 12 094 employees for five consecutive years during the study period. The number and mean age of participants in 2013 were 7568 (4323 men and 3245 women) and 43.7 (SD 8.3) years. The number of observations for each year is shown in table 1. The excluded 26 933 observations tended to be somewhat younger at the time of their exclusion (mean age: 34.0 versus 44.3 years), but the proportion of men was similar to those without missing variables (55.7% versus 58.1%). We also found that the excluded sample was somewhat healthier than the analytic sample (eg, 22.4 kg/m^2^ versus 23.1 kg/m^2^ for BMI and 112.2 mg/dl versus 120.6 mg/dl for LDL-C). The characteristics of the excluded sample are shown in the supplementary material (www.sjweh.fi/article/3966), table S1. All the employees included in our study consented to provide their information to our research group. The ethics committee at the Graduate School of Engineering, University of Tokyo approved this study (no. KE18-44, approved on November 6, 2018).

**Table 1:** Number of observations (obs) at annual health checkup of each year by sex and class.

Year	Sex		Class	
	
	Men	Women	Non-manager	Manager
			
	(Obs=26 648)	(Obs=19 240)	(Obs=30 390)	(Obs=15 498)
2013	4323	3245	4986	2582
2014	4711	3440	5379	2772
2015	5566	4017	6389	3194
2016	5897	4185	6707	3375
2017	6151	4353	6929	3575

### Measures

#### Managerial position

The status of being a manager (including executive and middle managers) was the exposure of our study. From the occupational class variable in the registration data, we identified whether each employee was a manager or not in each of the study years.

#### CVD risk factors

We obtained two types of CVD risk factors as outcomes from the annual health checkup data: metabolic risks and health-related behaviors. Metabolic risks were measured as seven laboratory variables and two body metrics, which were extracted from the annual health checkup data. The laboratory variables included systolic blood pressure (mmHg), diastolic blood pressure (mmHg), fasting blood sugar (mg/dl), HbA1c (%), LDL-C (mg/dl), HDL-C (mg/dl), and TG (mg/dl). The body metrics were BMI (kg/m^2^) and abdominal circumference (cm). Under the Japan’s health checkup schema, blood tests were conducted under accuracy control. Health professionals measured blood pressure and abdominal circumference using the standardized protocol set regulated by the government (22).

For health-related behaviors, we included binary answers to annual health checkup questionnaires regarding smoking habit [yes/no answers to “Do you habitually smoke? (You habitually smoke if you have ever smoked over 100 cigarettes in total or for six months or more and have also smoked in the last month.)”], exercise habits (yes/no answers to “Do you exercise more than 30 minutes, more than twice in a week and have continued this exercise habit for more than one year?”), and self-reported sufficiency of sleep (yes/no answers to “Getting enough sleep?”). We included the self-reported sufficiency of sleep as a CVD risk factor because of a previous study’s suggestion that self-reported lack of sleep was associated with impaired glucose tolerance (23).

### Covariates

We extracted the following covariates from the registration data: age as a categorical variable (30–39, 40–49, 50–59, or 60–69 years old), sex, average overtime-working hours as a continuous variable (hours per month, calculated for each of the fiscal years), years of employment as a categorical variable (<10, 10–19, 20–29, 30–39, or ≥40 years), and marital status as a categorical variable (currently married or not).

Besides these covariates, we examined whether each employee experienced hospitalization in each year throughout the study period from the claims data because hospitalization could be a confounder in our analyses (while individuals who were hospitalized might be urged to have better health-related behaviors, they would be less likely to be promoted due to leave of work during the hospitalization). In the main analyses, we did not adjust for this variable because (i) the incidence of hospitalizations could also be a mediator (eg, busy managers might not have enough time to be hospitalized and undergo test, resulting in deteriorated health) and (ii) the incidence of hospitalizations was rare in the study population (around 4%) as shown in table 2, similar to the general population (working age) (24). We adjusted for hospitalizations in the secondary analyses.

**Table 2:** Basic characteristics among employees registered through the study period ^a, b^ [Obs=observations; SD=standard deviation; BMI=body mass index; BP=blood pressure; LDL-C=low-density lipoprotein cholesterol; HDL-C=high-density lipoprotein cholesterol; TG=triglycerides].

Characteristics	Sex					Class				
	
	Men (Obs=26 648)		Women (Obs=19 240)		P Value ^c^	Non-manager (Obs=30 390)		Manager (Obs=15 498)		P Value ^c^
			
	%	Mean (SD)	%	Mean (SD)		%	Mean (SD)	%	Mean (SD)	
Age (years)										
30–39	33.5		33.3		<0.001	41.8		17.0		<0.001
40–49	32.2		41.9			32.5		43.7		
50–59	29.1		19.4			17.7		39.4		
60–69	5.2		5.4			8.0		0.0		
Sex										
Men	100		0		<0.001	40.4		92.7		<0.001
Women	0		100			59.6		7.3		
Overtime-work, hours/month		38.0 (22.6)		20.5 (16.7)	<0.001		22.6 (17.7)		46.5 (21.3)	<0.001
Years of employment										
<10	30.8		33.2		<0.001	39.8		16.0		<0.001
10–20	24.2		29.6			28.5		22.5		
20–30	31.4		28.8			22.7		45.2		
30–40	12.4		7.9			7.6		16.2		
>40	1.2		0.5			1.4		0.1		
Marital status										
Not married	12.8		52.5		<0.001	38.4		11.9		<0.001
Married	87.2		47.5			61.6		88.1		
Smoking habit										
Current	29.7		8.5		<0.001	17.6		27.1		<0.001
Not currently	70.3		91.5			82.4		72.9		
Exercise habit										
Yes	38.8		27.1		<0.001	32.3		37.1		<0.001
No	61.2		72.9			67.7		62.9		
Sufficient sleep										
Enough	62.4		53.8		<0.001	58.5		59.3		0.108
Not enough	37.6		46.2			41.5		40.7		
BMI, kg/m^2^		24.3 (3.4)		21.4 (3.4)	<0.001		22.5 (3.8)		24.2 (3.3)	<0.001
Abdominal circumference, cm		85.8 (9.2)		76.6 (9.3)	<0.001		80.2 (10.3)		85.4 (9.3)	<0.001
Systolic BP, mmHg		118.7 (14.9)		109.0 (15.1)	<0.001		112.8 (15.7)		118.1 (15.0)	<0.001
Diastolic BP, mmHg		75.6 (11.8)		67.8 (11.3)	<0.001		70.5 (11.9)		75.9 (11.9)	<0.001
Fasting blood sugar, mg/dl		99.7 (17.9)		92.2 (12.2)	<0.001		94.6 (14.3)		100.5 (18.7)	<0.001
HbA1c, %		5.6 (0.6)		5.4 (0.4)	<0.001		5.5 (0.5)		5.6 (0.6)	<0.001
LDL-C, mg/dl		125.4 (30.9)		114.0 (30.2)	<0.001		118.3 (31.0)		125.2 (30.9)	<0.001
HDL-C, mg/dl		57.1 (14.3)		72.2 (16.2)	<0.001		65.8 (17.1)		58.7 (15.2)	<0.001
TG, mg/dl		127.5 (104.0)		74.2 (48.5)	<0.001		94.2 (78.6)		126.5 (103.7)	<0.001
Hospitalization ^d^										
No	96.5		94.9		<0.001	95.5		96.4		<0.001
Yes	3.5		5.1			4.5		3.6		

a For the study period, 12 094 employees were included in this study (4323 men and 3245 women; age mean [SD]: 43.7 [8.3] years in 2013).

b The numbers of employees included were: 7568 in 2013, 8151 in 2014, 9583 in 2015, 10 082 in 2016, and 10 504 in 2017.

c P values were derived using t-test for overtime work, BMI, abdominal circumference, systolic BP, diastolic BP, fasting blood sugar, HbA1c, LDL-C, HDL-C, and TG. Pearson’s chi-squared test was applied to the other variables.

d Figures indicate the number of observations where an employee was hospitalized during each of the fiscal years.

### Statistical analysis

We first assessed the association between the status of being a manager and CVD risk factors using a pooled cross-sectional approach. We adopted a linear model specification for both the metabolic risk factors (continuous value outcomes) and the health-related behaviors, which took a dichotomous value (linear probability model: LPM). This LPM specification facilitated interpretation; that is, the coefficients, if multiplied by 100, showed the difference in the percentages of having each health-related behavior between managers and non-managers. Furthermore, it has been advocated that the marginal treatment effect derived by LPM is comparable to those based on models with better fit (25). We adjusted for age (in ten-year increments), sex, years of employment (in ten-year increments), and marital status in this analysis. We used heteroskedasticity-robust standard errors, HC3 (26).

Next, to adjust for individual time-invariant factors, we applied a fixed-effects model for each outcome variable. We supposed the following specification:


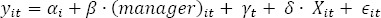


where *i* and *t* denoted individual and year, respectively. *y_it_* represented each of the outcomes measured for employee *i* in fiscal year *t*. *α_i_* was the individual time-invariant fixed-effects, whether they were observed or not. The individual fixed-effects (*α_i_*) accounted for both the observed trait, such as sex, and unobserved individual traits, such as time discount rate and education. *(manager)_it_* was a dummy variable, which took one if employee *i* was a manager in the fiscal year *t*, and took zero otherwise. *γ_t_* represented the year fixed-effects. *X*_it_ included exogenous time-varying covariates: age (in ten-year increments), years of employment (in ten-year increments), and marital status.

Finally, in order to account for the possibility that the impact of being a manager could differ depending on whether employees were “promoted” from non-manager to manager or “demoted” from manager to non-manager, we adopted an asymmetric fixed-effects model (27). In this analysis, we aimed to distinguish the effect of being “promoted” from being “demoted” through different coefficients in the model. In contrast, the traditional fixed-effects model assumes that these two effects are symmetric and thus represented by an identical coefficient. The supplementary appendix presents the model specification for the asymmetric fixed-effects analysis.

P-values <0.05 were statistically significant. We used R 3.6.2 for all analyses in this study (28). The “plm” package in R was used for the analyses (29).

### Secondary analysis

We conducted several secondary analyses. First, we additionally adjusted for average overtime-working hours in fiscal year *t*. Because employees’ work time may depend on whether or not they are managers, we added average overtime-working hours to the covariates to separate the association between a managerial position and our outcomes from the association mediated through overtime-working hours. Second, we additionally adjusted for the incidence of hospitalization in the year *t*. Third, we performed cross-sectional and fixed-effects analyses with the stratification according to each employee’s sex because the association between occupational class and CVD risk factors may differ according to sex. We preliminarily analyzed the pooled cross-sectional data with the interaction term between being a manager and sex; the interaction was statistically significant for some outcomes, such as TG and self-reported sufficiency of sleep. Moreover, a previous study revealed that a lower occupational class was associated with poor mental functioning among male workers, but not among female workers (30).

## Results

Among the 45 888 observations (covering 12 094 employees) registered over the fiscal years 2013–2017, 15 498 observations (33.8%) were in a managerial position (table 2). Overall, observations with manager status were more likely to involve men than those without manager status (92.7% versus 40.4%) and displayed higher levels for blood pressure, LDL-C and TG, fasting blood sugar, HbA1c, BMI, abdominal circumference, and current smoking habit. Managers were also more likely to have regular exercise habits. For our analyses, almost 80% of the 12 094 employees contributed to ≥3 of 5 observations (supplementary table S2).

### Metabolic risks

Table 3 illustrates the associations between being a manager and various metabolic risks. In the pooled cross-sectional analyses, managers’ BMI was 0.2 kg/m^2^ lower [95% confidence interval (CI) 0.0–0.3], systolic blood pressure was 1.4 mmHg lower (95% CI 0.8–2.1), and LDL-C level was 2.0 mg/dl lower (95% CI 0.7–3.3). However, they had a 1.0 mg/dl (95% CI 0.3–1.6) higher fasting blood sugar level. After adjusting for employee-level fixed effects, being a manager was associated with a 2.2 mg/dl (95% CI 0.8–3.7) significantly higher LDL-C level. The association between manager status and BMI, blood pressure, or fasting blood sugar was not significant.

**Table 3:** Association between being a manager and metabolic risks. [BMI=body mass index; BP=blood pressure; LDL-C=low-density lipoprotein cholesterol; HDL-C= high-density lipoprotein cholesterol; TG=triglycerides; CI=confidence interval]

	Pooled cross-sectional				Fixed-effects			
	
	Model 1 ^a^		Model 2 ^b^		Model 3 ^c^		Model 4 ^d^	
			
	Estimates ^e^	95% CI	Estimates ^e^	95% CI	Estimates ^e^	95% CI	Estimates ^e^	95% CI
BMI, kg/m^2^	-0.2 ^f^	-0.3–0.0	-0.2 ^f^	-0.4–0.0	0.0	0.0–0.1	0.0	-0.1–0.1
Abdominal circumference, cm	-0.2	-0.6–0.2	-0.1	-0.6–0.3	0.2	-0.1–0.4	0.1	-0.1–0.4
Systolic BP, mmHg	-1.4 ^g^	-2.1–-0.8	-1.2 ^g^	-1.8–-0.6	-0.1	-0.8–0.7	-0.1	-0.9–0.6
Diastolic BP, mmHg	-0.2	-0.6–0.3	0.0	-0.5–0.5	0.3	-0.2–0.9	0.3	-0.3–0.8
Fasting blood sugar, mg/dl	1.0 ^h^	0.3–1.6	1.1 ^h^	0.4–1.8	0.2	-0.4–0.9	0.2	-0.5–0.9
HbA1c, %	0.0	0.0–0.0	0.0	0.0–0.0	0.0	0.0–0.0	0.0	0.0–0.0
LDL-C, mg/dl	-2.0 ^h^	-3.3–-0.7	-2.5 ^g^	-3.9–-1.1	2.2 ^h^	0.8–3.7	2.2 ^h^	0.7–3.6
HDL-C, mg/dl	0.1	-0.6–0.8	0.3	-0.4–1.0	0.4	-0.1–0.9	0.4	-0.1–1.0
TG, mg/dl	2.1	-1.4–5.5	3.1	0.7–6.8	3.3	-1.0–7.7	2.8	-1.5–7.2

a We adjusted for age (in ten-year increments), sex, marital status, and years of employment (in ten-year increments) in Model 1.

b In Model 2, we adjusted for the covariates in Model 1 plus average overtime-working hours.

c We adjusted for age (in ten-year increments), marital status, and years of employment (in ten-year increments) in Model 3. We omitted the sex variable because we included individual time-invariant fixed-effects in the fixed-effects models.

d In Model 4, we adjusted for the covariates in Model 3 plus average overtime-working hours.

e Estimates indicate additive effects of being a manager on outcomes.

f P-value <0.05.

g P-value <0.001.

h P-value <0.01.

### Health-related behaviors

Table 4 demonstrates the association between being a manager and health-related behaviors. In the fixed-effects model, managers were 5.5% (95% CI 2.5–8.5) less likely to exercise regularly and reported getting enough sleep 6.1% less (95% CI 3.0–9.1) than non-managers, even though the pooled cross-sectional analysis did not show significant associations for these outcomes (point estimate, 0.7%; 95% CI -1.1–2.5 for exercise habits, and point estimate, -0.9%; 95% CI -2.8 –1.0, for self-reported sufficiency of sleep).

**Table 4:** Association between being a manager and health-related behaviors. [CI=confidence interval]

	Pooled cross-sectional				Fixed-effects			
	
	Model 1 ^a^		Model 2 ^b^		Model 3 ^c^		Model 4 ^d^	
			
	Estimates ^e^	95% CI	Estimates ^e^	95% CI	Estimates ^e^	95% CI	Estimates ^e^	95% CI
Smoking habit								
Current smoker	-1.8	-3.7–0.1	-1.5	-3.5–0.5	0.8	-0.7–2.3	0.6	-0.9–2.1
Exercise habit								
Exercise regularly	0.7	-1.1–2.5	2.3 ^f^	0.4–4.2	-5.5 ^g^	-8.5– -2.5	-4.6 ^h^	-7.6– -1.5
Sleep status								
Sleep enough	-0.9	-2.8–1.0	5.9 ^g^	3.9–7.9	-6.1 ^g^	-9.1– -3.0	-3.8 ^f^	-6.8– -0.9

a We adjusted for age (in ten-year increments), sex, marital status, and years of employment (in ten-year increments) in Model 1.

b In Model 2, we adjusted for the covariates in Model 1 plus average overtime-working hours.

c We adjusted for age (in ten-year increments), marital status, and years of employment (in ten-year increments) in Model 3. We omitted the sex variable because we included individual time-invariant fixed-effects in the fixed-effects models.

d In Model 4, we adjusted for the covariates in Model 3 plus average overtime-working hours.

e Estimates indicate additive effects of being a manager on outcomes. We showed the coefficients multiplied by 100, which showed the difference in the percentages of having each health-related behavior between managers and non-managers (Null hypothesis: coefficient = 0).

f P-value <0.05.

g P-value <0.001.

h P-value <0.01.

### Asymmetric effects of manager status

Supplementary table S3 presents the number of observations regarding the occupational class changes during the study period. In our dataset, some employees changed their position from a manager to a non-manager, especially when they were transferred to another branch or department or turned 60. While there were 523 observations (1.6%) where employees were promoted from non-manager to manager (“promotion”), there were 154 observations (0.5%) where employees changed their status from manager to non-manager (“demotion”). When we focused on the asymmetric effects of manager status on CVD risk factors (asymmetric fixed-effects analyses), promotion to manager was associated with a 2.2 mg/dl (95% CI 0.1–4.3) higher LDL-C level and a 6.4% (95% CI 1.5–11.3) lower likelihood of engaging in regular exercise (tables 5 and 6).

**Table 5:** Association between being a manager and metabolic risks in the asymmetric fixed-effects model. [BMI=body mass index; BP=blood pressure; LDL-C=low-density lipoprotein cholesterol; HDL-C=high-density lipoprotein cholesterol; TG=triglycerides; CI=confidence interval].

	Promotion ^a^		De-promotion ^a^	
	
	Estimates ^b^	95% CI	Estimates ^b^	95% CI
BMI, kg/m^2^	0.0	-0.1–0.1	-0.1	-0.3–0.0
Abdominal circumference, cm	0.1	-0.3–0.5	-0.4	-1.0–0.3
Systolic BP, mmHg	-0.3	-1.4–0.8	-0.9	-2.7–0.9
Diastolic BP, mmHg	0.0	-0.8–0.8	-1.0	-2.3–0.2
Fasting blood sugar, mg/dl	0.1	-0.7–1.0	0.6	-1.9–3.1
HbA1c, %	0.0	0.0–0.0	0.0	-0.1–0.0
LDL-C, mg/dl	2.2 ^c^	0.1–4.3	-0.5	-3.6–2.6
HDL-C, mg/dl	0.4	-0.3–1.2	-0.3	-1.5–1.0
TG, mg/dl	4.0	-3.4–11.4	-6.1	-18.8–6.6

a We adjusted for age (in ten-year increments), marital status, and years of employment (in ten-year increments). We omitted the sex variable because we included individual time-invariant fixed-effects in this model.

b Estimates indicate additive effects of being a manager on outcomes.

c P-value <0.05.

**Table 6:** Association between being a manager and health-related behaviors in the asymmetric fixed-effects model. [CI=confidence interval]

	Promotion ^a^		De-promotion ^a^	
	
	Estimates ^b^	95% CI	Estimates ^b^	95% CI
Current smoker	-0.5	-2.7–1.7	-3.2	-6.5–0.1
Exercises regularly	-6.4 ^c^	-11.3– -1.5	8.6 ^c^	1.2–16.0
Sleeps enough	-2.2	-6.9–2.6	22.5 ^d^	14.9–30.1

a We adjusted for age (in ten-year increments), marital status, and years of employment (in ten-year increments). We omitted the sex variable because we included individual time-invariant fixed-effects in this model.

b Estimates indicate additive effects of being a manager on outcomes. We showed the coefficients multiplied by 100, which showed the difference in the percentages of having each health-related behavior between managers and non-managers (Null hypothesis: coefficient = 0).

c P-value <0.05 and

d P-value <0.001.

### Secondary analyses

The results were qualitatively unaffected when adjusted for the average overtime-working hours for both the pooled cross-sectional analyses and the fixed-effects model (tables 3 and 4). Our findings were unaffected after further adjustment for the incidence of any claims-identified hospitalization in the year, suggesting that hospitalization had little impact on our conclusions (supplementary tables S4 and S5). When we performed pooled cross-sectional and fixed-effects analyses stratifying by sex, similar associations of being a manager with LDL-C level, exercise habit, and self-reported sufficiency of sleep were observed among male employees. In the female subsample, a statistically significant association was not observed except for self-reported sufficiency of sleep in the pooled cross-sectional analysis (supplementary tablesS6 and S7).

## Discussion

This study used unique panel data that included employees’ occupational class and annual health checkup data. Using fixed-effects models to adjust for time-invariant employee characteristics, we found that managers demonstrated higher levels of CVD risk factors These results were in contrast to the findings observed when we did not adjust for employees’ fixed-effects (ie, pooled cross-sectional analyses), suggesting the possibility that unobserved individual characteristics might confound the relationship between manager status and health status. Our findings imply that being a manager could have a negative impact on employees’ health, even though managers seemed to be healthier than non-managers in the pooled cross-sectional analyses.

Two possible mechanisms may explain our results. First, we could refer to two mainstream frameworks in the context of job stress: the job demand–control (JDC) model and the effort–reward imbalance (ERI) model (31, 32). These models maintain that job stress is determined by the joint effects of job demand and job control (JDC model) or the balance between employees’ efforts and rewards for their job (ERI model). Thus, the models can infer that being a manager can be stressful when the job is demanding or involves insufficient rewards. Job stress, in turn, has been reported to be associated with metabolic syndrome, physical inactivity, and sleep disturbance (8, 33, 34). Overall, these studies support our hypothesis that the status of a manager could contribute to CVD risk factors due to increased job stress. Our analyses are also consistent with these job stress models. In the fixed-effects analyses, the inverse associations of being a manager with LDL-C level, exercise habits, and self-reported sufficiency of sleep persisted even after adjusting for the overtime-working hours. This result is reasonable under the JDC or ERI models in the sense that job stress is not determined only through how many hours employees may work but reflects the joint effects of job demand and control or the balance between employees’ efforts and rewards for their job.

Second, it is possible that being a manager could be associated with reducing employees’ free time outside of work. According to the Grossman model, people must invest time to produce health (35). If employees in a managerial position lack sufficient time to pay attention to a healthy diet, engage in regular exercise, or sleep sufficiently because of their working environment, being a manager could cause unfavorable health outcomes. Our asymmetric fixed-effects models showed that promotion to a manager was associated with a higher LDL-C level and a lower likelihood of regular exercise and sufficient sleep. These results were consistent with our main results from the fixed-effects model, supporting our hypothesis that differing working environments between managers and non-managers can alter the way employees use their time. Overall, employers may have the potential to avoid workers’ CVD risks by paying attention to their stress status and working environment.

In contrast to the analysis adjusting for employees’ fixed-effects, the pooled cross-sectional analysis showed that managers had *lower* levels of CVD risk factors, such as BMI, systolic blood pressure, and LDL-C, than non-managers. However, these associations were inverse for LDL-C level, exercise habits, and self-reported sufficiency of sleep in the fixed-effects models. Since the fixed-effects models accounted for employees’ time-invariant traits and reduced the bias derived from individual-level unobserved characteristics, we could claim that the true associations between managerial status and the reported health outcomes should be inverse, and the positive associations observed in our cross-sectional analyses were specious.

Our study was built upon prior studies investigating the relationship between occupational class and health conditions. In Western countries, previous cross-sectional or cohort studies revealed that managers were better off than non-managers in terms of heart disease and health-related behaviors (9, 10, 13), as our pooled cross-sectional results also suggested. However, only four longitudinal studies addressed the possibility that employees with some unobserved individual traits were more likely to be promoted (16–19). While one study suggested positive impacts of promotion on heart disease (16), the other three studies demonstrated that promotion to a manager was associated with poorer mental health, possibly due to job stress (17–19). Our study is consistent with the latter three studies and has added credible evidence to the literature by taking into account employee-level fixed-effects, whether observable or not. Our study also extends these previous findings to other contexts, such as Asian countries, because the role of occupational class in terms of impact on health may differ between Western and Asian countries (36). It has been reported that a managerial position was associated with better health in Western countries, but the association was in the opposite direction in Asian countries. Several cross-sectional studies have shown that, contrary to Western countries, managerial employees in Japan and South Korea exhibited higher mortality than other workers, although suicide or cancer, instead of CVD, were the main factors contributing to higher mortality among managerial employees (20, 36). Another cross-sectional study revealed that Japanese non-manual (higher-grade) employees had lower HDL-C and higher BMI than manual (lower-grade) employees (37). Our results are consistent with those studies in the sense that higher-grade workers experienced unfavorable health outcomes.

This study also contributed to the evidence of health inequality in Japan. Compared with Western countries, the health inequality in Japan had not been strongly expressed (38). This contrast could be attributable to Japan’s egalitarian society, represented by its equal education opportunities and financial access to healthcare (39). However, it has been pointed out that globalization and rising economic disparity can accelerate the health inequality even in Japan (39); several studies indicated that Japan’s economic stagnation might cause mortality inequality among workers (20, 36). In this context, our study has added evidence on Japan’s health inequality among workers in terms of CVD risk factors. Further studies are expected to unravel the whole picture of health inequality in Japan.

Some limitations of this study should be acknowledged. First, our study was observational, and we failed to obtain information on education and unobserved health conditions, which could be confounders for the association between a managerial position and CVD risks. The association in the pooled cross-sectional analyses might be confounded, as education has been shown to be associated with CVD risks (40). Nevertheless, the individual fixed-effects enabled us to avoid the confounding by education because education was reasonably an individual-specific time-invariant factor in our research. As for unobserved health conditions, healthier employees might be more likely to be promoted to a manager, as previous studies suggested (17, 41). However, in such a case, we would expect a bias toward the null, and the extent to which managers experienced poorer health status would be larger than our estimates. Moreover, detrimental health status that could affect promotion seemed rare in our study subjects; only a small part of the employees experienced hospitalization (3.6% and 4.5% among managers and non-managers, respectively) (table 2). Second, we did not identify the mechanisms through which manager status was associated with higher levels of CVD risk factors because we did not have data for the JDC or ERI models. Further research is expected to reveal exact mechanisms. Third, most of the managers in this study were male. Because a previous study revealed that the association of a lower occupational class with poor mental functioning varied according to sex (30), the association between occupational class and CVD risk factors may differ according to employee sex. This possible sex-heterogeneity, however, could not be examined sufficiently because of the small proportion of female managers in our dataset. To evaluate the sex-heterogeneity, further studies with a dataset including more female managers and non-managers are expected. Finally, we had access to only one Japanese company’s data and restricted study subjects to employees with health checkup data. Moreover, our results should be taken with caution for younger workers because the excluded 26 933 observations due to missing variables were somewhat younger and healthier than those included in our analyses. Another concern for generalizability is that the questionnaires for health-related behaviors were just binary and crude.

Even with these concerns about generalizability, we believe that this study is valuable, as it presents the possibility that being a manager could have a negative effect on employees’ health, especially among male Japanese employees, contrary to the conclusions found in most of the previous literature.

This study has two main strengths. First, we used repeated measures of occupational class and health conditions. The data allowed us to account for time-invariant unobserved individual characteristics, in contrast to the previous studies that might not completely account for individual characteristics. Second, we examined the association between a managerial position and a wide variety of CVD risks using data for annual health checkup data in Japan. This extensive wide variety allowed us to evaluate metabolic risks as well as health-related behaviors.

### Concluding remarks

We found that managers had higher levels of CVD risk factors in multiple dimensions (LDL-C, exercise habits, and self-reported sufficiency of sleep) compared to non-managers after adjusting for employee-level observed and unobserved time-invariant characteristics. These unfavorable health risks associated with promotion to a manager should be considered in occupational settings.

## Supplementary material

Supplementary material
