# Supplementary material for: Association of managerial position with cardiovascular risk factors: A fixed-effects analysis for Japanese employees
Source: Scand J Work Environ Health. 2021 Aug 31;47(6):425–34. doi: 10.5271/sjweh.3966 (PMC8504547; doi:10.5271/sjweh.3966)
Supplement: Supplementary material [file SJWEH-47-425-S001.pdf]

Association of managerial position with cardiovascular risk factors: A fixed-effects analysis for Japanese employees[1]

by Ryo Ikesu, MD, Atsushi Miyawaki, PhD, Akiko Kishi Svensson, PhD,[2] Thomas Svensson, PhD, Yasuki Kobayashi, PhD, Yuichi Tei/Ung-il Chung, PhD

1. *Supplementary material*
2. *Correspondence to: Akiko Kishi Svensson, PhD, Precision Health, Department of Bioengineering, Graduate School of Engineering, The University of Tokyo, 7-3-1 Hongo, Bunkyo-ku, Tokyo 113-0033, Japan. [E-mail: akiko-kishi@umin.ac.jp]*

Appendix

We adopted the asymmetric fixed-effects model, based on the specification mentioned in a previous study (27).
The model specification was:
$y_{it} = \alpha_i + \beta^+ \cdot Z_{it}{}^+ + \beta^- \cdot Z_{it}{}^- + \gamma_t + \delta \cdot X_{it} + \epsilon_{it}$,
where

$$Z_{it}{}^+ = \sum_s (manager)_{is}{}^+$$

$$Z_{it}{}^- = \sum_s (manager)_{is}{}^-$$

i and *t* denoted individual and year, respectively. y_{it} represented each of the outcomes measured for employee *i* in the fiscal year *t*. $(manager)_{it}{}^+$ was a dummy variable, which took one if employee *i* was a manager in the fiscal year *t* but not in *t-1*, and took zero otherwise. $(manager)_{it}{}^-$ was a dummy variable, which took one if employee *i* was not a manager in the fiscal year *t* but was in *t-1*, and took zero otherwise. As in the main fixed-effects analyses, X_{it}, α_i, γ_t, ϵ_{it} were exogenous time-varying covariates, the individual time-invariant fixed-effects, the year fixed-effects, and an idiosyncratic error term, respectively. With this specification, we estimated the association of CVD risk factors with "promotion" to manager and the association with "demotion" to non-manager, through β^+ and β^-, respectively.

Table S1 Characteristics of the included and excluded sample.

Characteristics		Included sample (Obs. = 45 888)	Excluded sample (Obs. = 26 933)
Age, mean (SD)		44.3 (8.8)	34.0 (11.6)
Sex, %	Men	58.1	55.7
	Women	41.9	44.3
Smoking habit, %	Current	20.8	23.5
	Not currently	79.2	76.5
Exercise habit, %	Yes	33.9	41.1
	No	66.1	58.9
Sleep status, %	Enough	58.8	60.2
	Not enough	41.2	39.8
BMI, mean (SD), kg/m2		23.1 (3.7)	22.4 (3.6)
Abdominal circumference, mean (SD), cm		81.9 (10.3)	78.7 (10.5)
Systolic BP, mean (SD), mmHg		114.6 (15.7)	112.5 (14.2)
Diastolic BP, mean (SD), mmHg		72.3 (12.2)	68.0 (10.7)
Fasting blood sugar, mean (SD), mg/dl		96.6 (16.2)	91.7 (14.3)
HbA1c, mean (SD), %		5.5 (0.6)	5.5 (0.6)
LDL-C, mean (SD), mg/dl		120.6 (31.1)	112.2 (30.6)
HDL-C, mean (SD), mg/dl		63.4 (16.8)	63.4 (16.4)
TG, mean (SD), mg/dl		105.1 (89.2)	92.7 (74.1)
Hospitalization, %	No	95.8	94.4
	Yes	4.2	5.6

SD: standard deviation. BMI: body mass index. BP: blood pressure. LDL-C: low-density lipoprotein cholesterol. HDL-C: high-density lipoprotein cholesterol. TG: triglycerides.

Table S2 Number of observations each employee contributed.

No. of observation(s)	Proportion (%) (N = 12 094)
1	10.9
2	10.6
3	14.7
4	16.0
5	47.8

Table S3 Number of observations in occupational-class changes.

	Observation	Proportion (%)
Non-manager to non-manager	20 861	64.1
Non-manager to manager	523	1.6
Manager to non-manager	154	0.5
Manager to manager	11 013	33.8
Total[a]	32 551	100.0

[a] The number of total observations in this table is not equal to the number of observations included in our main analyses, because we omitted observations that did not have information on an occupational class in the previous year.

Table S4 Association between being a manager and metabolic risks with the adjustment for hospitalization in the year.

	Pooled cross-sectional[a]		Fixed-effects[b]	
	Estimates	95% CI	Estimates	95% CI
BMI, kg/m2	-0.2*	-0.3–0.0	0.0	0.0–0.1
Abdominal circumference, cm	-0.2	-0.6–0.2	0.2	-0.1–0.4
Systolic BP, mmHg	-1.4***	-2.1–-0.8	-0.1	-0.8–0.7
Diastolic BP, mmHg	-0.2	-0.6–0.3	0.3	-0.2–0.9
Fasting blood sugar, mg/dl	1.0**	0.3–1.6	0.2	-0.4–0.9
HbA1c, %	0.0	0.0–0.0	0.0	0.0–0.0
LDL-C, mg/dl	-2.0**	-3.3–-0.7	2.2**	0.8–3.7
HDL-C, mg/dl	0.1	-0.6–0.8	0.4	-0.1–0.9
TG, mg/dl	2.1	-1.4–5.6	3.3	-1.0–7.7

BMI: body mass index. BP: blood pressure. LDL-C: low-density lipoprotein cholesterol. HDL-C: high-density lipoprotein cholesterol. TG: triglycerides. CI: confidence interval. Estimates indicate additive effects of being a manager on outcomes. * denotes p-value < 0.05, ** denotes p-value < 0.01, and *** denotes p-value < 0.001.

[a] We adjusted for age (in ten-year increments), sex, marital status, years of employment (in ten-year increments), and hospitalization in the year.

[b] We adjusted for age (in ten-year increments), marital status, years of employment (in ten-year increments), and hospitalization in the year. We omitted the sex variable because we included individual time-invariant fixed-effects in the fixed-effects model.

Table S5 Association between being a manager and health-related behaviors with the adjustment for hospitalization in the year.

	Pooled cross-sectional[a]		Fixed-effects[b]	
	Estimates	95% CI	Estimates	95% CI
Smoking habit (Current smoker)	-1.8	-3.7–0.1	0.8	-0.7–2.3
Exercise habit (Exercise regularly)	0.7	-1.1–2.5	-5.5***	-8.6–-2.5
Sleep status (Sleep enough)	-0.9	-2.8–1.0	-6.1***	-9.1–-3.0

CI: confidence interval. Estimates indicate additive effects of being a manager on outcomes. We showed the coefficients multiplied by 100, which showed the difference in the percentages of having each health-related behavior between managers and non-managers (Null hypothesis: coefficient = 0). *** denotes p-value < 0.001.

[a] We adjusted for age (in ten-year increments), sex, marital status, years of employment (in ten-year increments), and hospitalization in the year.
[b] We adjusted for age (in ten-year increments), marital status, years of employment (in ten-year increments), and hospitalization in the year. We omitted the sex variable because we included individual time-invariant fixed-effects in the fixed-effects model.

Table S6 Association between being a manager and metabolic risks (stratification: sex).

	Men				Women			
	Pooled cross-sectional		Fixed-effects		Pooled cross-sectional		Fixed-effects	
	Estimates	95% CI	Estimates	95% CI	Estimates	95% CI	Estimates	95% CI
BMI, kg/m2	-0.2*	-0.4–0.0	0.0	0.0–0.1	-0.1	-0.5–0.2	0.0	-0.1–0.2
Abdominal circumference, cm	-0.2	-0.7–0.3	0.2	-0.1–0.5	-0.4	-1.3–0.6	0.3	-0.5–1.0
Systolic BP, mmHg	-1.8***	-2.5–-1.1	-0.2	-1.0–0.6	-0.7	-2.2–0.8	0.1	-1.6–1.9
Diastolic BP, mmHg	-1.0***	-1.5–-0.4	0.3	-0.3–0.9	0.3	-0.8–1.4	0.2	-1.1–1.4
Fasting blood sugar, mg/dl	0.2	-0.7–1.0	0.3	-0.5–1.1	0.5	-0.7–1.8	0.2	-0.9–1.3
HbA1c, %	0.0	0.0–0.0	0.0	0.0–0.0	0.0	0.0–0.1	0.0	0.0–0.0
LDL-C, mg/dl	-1.0	-2.6–0.6	2.9***	1.3–4.5	-0.6	-3.6–2.3	0.3	-2.6–3.2
HDL-C, mg/dl	-0.1	-0.8–0.7	0.4	-0.1–0.9	1.2	-0.5–2.9	0.0	-1.5–1.5
TG, mg/dl	-0.1	-4.4–4.2	3.7	-1.5–8.9	-3.6	-7.5–0.2	-0.3	-4.1–3.5

BMI: body mass index. BP: blood pressure. LDL-C: low-density lipoprotein cholesterol. HDL-C: high-density lipoprotein cholesterol. TG: triglycerides. CI: confidence interval. Estimates indicate additive effects of being a manager on outcomes. * denotes p-value < 0.05 and *** denotes p-value < 0.001.

We adjusted for age (in ten-year increments), marital status, and years of employment (in ten-year increments) in the pooled cross-sectional analysis.

Table S7 Association between being a manager and health-related behaviors (stratification: sex).

	Men				Women			
	Pooled cross-sectional		Fixed-effects		Pooled cross-sectional		Fixed-effects	
	Estimates	95% CI	Estimates	95% CI	Estimates	95% CI	Estimates	95% CI
Smoking habit (Current smoker)	-2.9*	-5.3–-0.5	1.2	-0.6–3.0	-1.1	-4.2–1.9	0.0	-1.1–0.9
Exercise habit (Exercise regularly)	-0.2	-2.4–2.0	-5.3**	-8.7–-2.0	1.5	-2.6–5.6	-5.5	-12.6–1.6
Sleep status (Sleep enough)	-4.5***	-6.7–-2.2	-6.9***	-10.3–-3.5	5.2*	0.6–9.8	-4.9	-11.6–1.8

CI: confidence interval. Estimates indicate additive effects of being a manager on outcomes. We showed the coefficients multiplied by 100, which showed the difference in the percentages of having each health-related behavior between managers and non-managers (Null hypothesis: coefficient = 0). * denotes p-value < 0.05, ** denotes p-value < 0.01, and *** denotes p-value < 0.001.

We adjusted for age (in ten-year increments), marital status, and years of employment (in ten-year increments) in the pooled cross-sectional analysis.
